# Severe Community-Acquired Pneumonia: Impact of HIV on Clinical Presentation, Microbiological and Laboratory Findings, and Outcome

**DOI:** 10.1177/08850666251359546

**Published:** 2025-07-16

**Authors:** JP Venturas, A Titus, GA Richards, C Feldman

**Affiliations:** 1 Department of Internal Medicine, Faculty of Health Sciences, University of the Witwatersrand Medical School, Johannesburg, South Africa; 2 Independent Statistician, Johannesburg, South Africa; 3 Dept of Surgery, Division of Critical Care, Faculty of Health Sciences, University of the Witwatersrand, Johannesburg, South Africa; 4 Department of Internal Medicine, Faculty of Health Sciences, University of the Witwatersrand, Johannesburg, South Africa

**Keywords:** human immunodeficiency virus, intensive care unit, laboratory findings, microbial aetiology, radiographic findings, severe community-acquired pneumonia, severity of illness, mortality

## Abstract

Severe community-acquired pneumonia (SCAP) is associated with significant morbidity and mortality, but there is a paucity of data regarding these infections in sub-Saharan Africa, especially among people living with HIV (PLWH). This study investigated the impact of HIV on clinical presentation, microbial aetiology, laboratory findings, and outcome of SCAP. This was additional analysis of data from a large, single-centre, retrospective, observational study conducted among consecutive adult patients (≥18 years) admitted to the multidisciplinary ICU at the Charlotte Maxeke Johannesburg Academic Hospital, between 1 July 2007 and 31 May 2019, with SCAP. The current study describes 718 PLWH and 131 HIV-negative cases extracted from the initial cohort. The median age was 37 [IQR 30-46] years with PLWH significantly younger than their HIV-negative counterparts (36 years [IQR 29-44] years vs 52 years [IQR 34-65] years; P < .001). PLWH were more commonly female (P = .053), while more of the HIV-negative patients were male. The median CD_4_ count of the PLWH was 42 [IQR 14-108] cells/mm^3^ and only 15.5% were on anti-retroviral therapy (ART) prior to hospitalisation. Differences were noted in clinical, laboratory and radiological features between the groups. Overall, *Mycobacterium tuberculosis* was the most common microbial aetiology in both groups, followed by *Streptococcus pneumoniae,* which was associated with a significantly lower mortality, whereas mortality with *Pneumocystis jirovecii* infection, which occurred only in PLWH, was high. Overall ICU mortality was high (48.9%), and while HIV was an independent risk factor for mortality (OR 0.58, 95% CI 0.37-0.92; p = .02) on univariate analysis, this finding was not true when HIV considered within the multivariable analysis. This study describes one of the largest cohorts of PLWH with SCAP and compares their findings with HIV-negative cases. HIV was not a significant predictor of mortality when considered in the context of other covariables on multivariable analysis.

## Introduction

The lung is one of the most frequently affected organ systems in people living with HIV (PLWH), commonly manifesting with infectious complications.^1^ Community-acquired pneumonia caused by *Streptococcus pneumoniae* (SP) remains the most common cause in most areas of the world, exceeded only by tuberculosis in sub-Saharan Africa and other low- middle-income countries (LMIC).^1,2^ Several studies have described CAP in hospitalised PLWH^2–16^ and although there were some similarities compared to HIV-negative patients, there were also significant differences. One unresolved question is what impact HIV has on patient outcomes. Some studies have suggested that the outcomes are the same in PLWH and HIV-negative patients, particularly among those that are virally-suppressed and have a CD4^+^ T-cell count > 350/mm^3.^^2,6,17–20^ while others have documented a higher mortality associated with HIV.^21,22^

There is no universally accepted definition of severe community-acquired pneumonia (SCAP), and the term is used most commonly in the literature to describe patients with CAP who require ICU admission.^23–29^ Other terms sometimes used in the description of these cases include multisystem disease and/or multiple organ failure; requiring intensive monitoring and/or organ supportive care; higher morbidity and mortality; increased use of healthcare resources; and/or the diagnosis is based on one or more severity of illness scores.^23–29^ Nearly one in five patients hospitalised with CAP require ICU admission, with mortality reaching > 25% within 30 days, and as high as 50% within one year following infection.^28^ Few studies or guidelines on SCAP have included PLWH and/or patients with various other forms of immunocompromise, who are at increased risk of infection with a broader spectrum of pathogens including *Mycobacterium tuberculosis* (MTB), *Pneumocystis jirovecii* (PJP), and other less common microorganisms.^29–31^ This is particularly so with regard to recent studies of SCAP in LMIC, such as sub-Saharan Africa and Asia, where few studies have compared PLWH with HIV-negative cases.^11,32–36^

The aim of this study was to describe a cohort of SCAP in PLWH, inclusive of all putative microbial aetiologies, and to compare all aspects of their infection, particularly mortality, with patients without HIV.

## Patients and Methods

This study was a further analysis of data from a large, single-centre, retrospective, observational study conducted among consecutive adult patients (≥ 18 years) admitted to the multidisciplinary ICU at the Charlotte Maxeke Johannesburg Academic Hospital (CMJAH), between 1 July 2007 and 31 May 2019, with a diagnosis of SCAP. The full details of the original study have been published elsewhere.^37^

The current study compared the demographic, clinical and laboratory findings among PLWH with those that were HIV-negative. The criteria used to confirm the diagnosis of CAP were the same as those described previously.^38^ The sources of data were ICU admission records, electronic discharge summaries and/or clinical case notes, and the Picture Archiving and Communication system (PACS; Phillips Intellispace PACS Radiology version 4.4.532.1), a pan-electronic platform used at CMJAH to access radiology.

Data collected included demographic and clinical details, severity of illness scores, laboratory data (including microbiology), radiological features, and details of the clinical course, ICU management, and patient outcomes. Data extraction was undertaken once approval had been obtained from the University of the Witwatersrand Human Research Ethics Committee (Medical) (Clearance certificate number M2008109) and the Chief Executive Officer of CMJAH.

Severity of illness was assessed using the CURB-65 score (Confusion, Urea >7 mmol/L, Respiratory rate ≥ 30 breaths/minute, Blood pressure < 90mm Hg (systolic) and/or ≤ 60mm Hg (diastolic), Age ≥ 65years^39^) and the Apache II score.^40,41^ In addition, the ratio of the partial pressure of oxygen in arterial blood to the fraction of inspired oxygen (PF) ratio, one of the minor criteria of severity of CAP as per the Infectious Disease Society of America/American Thoracic Society guideline, was calculated for each patient.^38^

Results of routine laboratory tests, performed for all patients on admission, were collected. For HIV testing, the Cobas HIV-1/HIV-2 qualitative assay (Roche) was used for screening, and HIV Ag/Ab combo assay (Abbott) for confirmation. HIV testing was performed with informed consent (in accordance with South African HIV testing policies at that time),^42^ together with the corresponding CD4 cell count (flow cytometry on a Beckman-Coulter MPLFC500 cytometer) and HIV viral load (HIVVL) (Roche Cobas^®^ 6800/8800 HIV-1 test). Routine microbiological testing was mostly in accordance with recommendations of the South African CAP guideline.^26^ Results of any additional, more specialised, microbiological testing undertaken at the discretion of the attending physician, were also collected.

Data analysis was performed using GraphPad Prism (v9) and Statistical Package for Social Sciences (SPSS) software, IBM manufacturer, Chicago, USA version 21.0. Continuous variables were presented as means and standard deviations or medians and interquartile ranges, as appropriate, and for comparison, unpaired t-tests or Mann-Whitney U tests were used. The categorical variables were presented as percentages, and associations among the categorical variables assessed using the Chi-squared or Fisher's exact tests. All tests were 2-tailed. Univariate and multivariable logistic regression analysis (MVA) with a generalized linear model were performed to identify which parameters were independent risk factors for mortality. Variables for the final multivariable model were backward selected, including variables with p > .20 on univariate analysis. However, HIV-specific variables were manually excluded, as data was not equally applicable to both the PLWH and HIV-negative groups. Additionally, variables with more than 10% missing data were manually excluded. Odds ratios for both univariate and multivariate modelling were reported. A P value of <.05 was regarded as statistically significant.

## Results

From the original study of 931 patients with SCAP, 718 were PLWH and 131 HIV-negative and were included into the current study of 849 patients of known HIV status.

The median age was 37 [IQR 30-46] years with PLWH significantly younger than the HIV-negative group (36 years [IQR 29-44] vs 52 years [IQR 34-65] years; P < .001), and a smaller proportion of PLWH were aged ≥65 years (17 (2.4%) versus 33 (25.2%) respectively; P < .001). More PLWH were female (387 (53.9%); P = .053), while more of the HIV-negative patients were male. Fewer PLWH were smokers (19 (2.6%) versus 14 (10.7%) in HIV-negative cases; P < .001).

Table 1 documents the comorbid conditions present. In PLWH, 35.5% had additional comorbidities other than HIV, compared to 66.4% in the HIV-negative group (P < .001). In the latter group, diabetes mellitus (DM), chronic cardiovascular (CVD), respiratory (including chronic obstructive pulmonary disease), and renal diseases were significantly more common (P < .001). More PLWH had a history of previous MTB infection.

**Table 1.** Co-Morbid Diseases and additional Conditions in PLWH and HIV-Negative Patients with SCAP (849 Patients).

Co-morbid disease, n	PLWH, n (%) (718 patients)	HIV-ve, n (%) (131 patients)	P value
Total, 342	255 (35.5%)	87 (66.4%)	**<0** **.** **001**
Chronic CVD, 115	77 (10.7%)	38 (29.0%)	**<0**.**001**
Chronic Respiratory disease, 64	35 (4.9%)	29 (22.1%)	**<0**.**001**
COPD, 34	14 (1.9%)	20 (15.3%)	**<0**.**001**
Diabetes mellitus, 51	27 (3.8%)	24 (18.3%)	**<0**.**001**
Previous MTB, 60	58 (8.1%)	2 (1.5%)	–
Pregnancy, 59	54 (7.5%)	5 (3.8%)	0.18
Current MTB, 31	29 (4.0%)	2 (1.5%)	–
Chronic kidney disease, 24	12 (1.7%)	12 (9.2%)	**<0**.**001**
Malignancy, 20	15 (2.1%)	5 (3.8%)	0.22

Statistical analysis using Fisher's Exact test.

Abbreviations: COPD: Chronic Obstructive Pulmonary Disease; CVD: cardiovascular disease; HIV: Human Immunodeficiency Virus; MTB: *Mycobacterium tuberculosis;* n: number; PLWH: people living with HIV; -ve: negative; %: percentage; Bold numbers indicate significant P values.

Table 2 documents the clinical features, severity scores, laboratory data and radiological findings of PLWH compared to HIV-negative cases. PLWH had higher median temperatures (P < .001), although the difference was numerically small. The median Apache II score was higher in PLWH (18 [IQR 14-23] vs 17 [IQR 12-21] in those that were HIV-negative; P = .02); however, there were no significant differences in the CURB-65 score, the PF ratio, nor in the proportion of patients with an Apache II score > 25 or in cases with a PF ratio ≤ 250.

**Table 2.** Comparison of Clinical Presentation, Laboratory Data and Chest Radiographic Finding in PLWH and HIV-Negative Patients with SCAP (849 Patients).

Parameter	PLWH	HIV-ve	P value
**Clinical features**	**median [IQR], n or n (%)**	**median [IQR], n or n (%)**	
Body temperature °C	36.9 [36.2-37.7], 699	36.5 [35.9-37], 128	**<0**.**001**
MAP mm Hg	79.3 [65.8-91.7], 683	80 [68-94], 125	0.72
Confusion	372 (52.4%)	71 (55.5%)	0.59
**SOI scores**	**median [IQR], n or n (%)**	**median [IQR], n or n (%)**	
CURB-65 score	2 [2-3], 692	3 [2-3], 126	0.06
Apache II score	18 [14-23], 669	17 [12-21], 124	**0**.**02**
Apache II score > 25	102 (15.3%)	16 (12.9%)	0.59
PF ratio	150 [96.7-230.3], 675	133.8 [105-206.1], 126	0.29
PF ratio ≤ 250	533 (79.0%)	106 (84.1%)	0.23
**Laboratory**	**median [IQR]**	**median [IQR]**	
Hb g/dL	10.2 [8.3-11.8], 713	11.7 [9.5-13.7], 130	**<0**.**001**
Hct %	0.3 [0.3-0.4], 713	0.4 [0.3-0.4], 130	**<0**.**001**
White cell count ×10^9^/L	9.1 [5.1-14.0], 712	13.3 [8.8-19.1], 129	**<0**.**001**
Platelet counts ×10^9^/L	206 [118-302.5], 711	220 [164-319],127	**0**.**02**
Sodium mmol/dL	135 [131-140], 714	138 [134-142], 130	**<0**.**001**
Potassium mmol/dL	4.3 [3.8-5], 714	4.6 [3.9-5.1], 130	**0**.**04**
Urea mmol/L	12 [6.1-23.5], 714	11.4 [6.8-21.7], 130	0.99
Creatinine μmol/L	124 [71-297], 713	128 [75.3-288], 130	0.87
CRP mg/L	195.5 [116-283], 676	209 [114.5-397], 127	0.52
Total protein g/L	64 [54-75.5], 687	59 [53.5-66.5],123	**0**.**001**
Albumin g/L	25 [21-29], 686	28 [25-34], 124	**<0**.**001**
Total bilirubin μmol/L	11 [6-23], 690	10 [6-18], 124	0.58
AST U/L	91[49-202.5], 690	58 [33-118.8], 124	**<0**.**001**
ALT U/L	48 [25-94.3], 688	39.5 [22.8-84.8], 124	0.17
ALP U/L	99 [70-152], 688	102.5 [73-133.3], 124	0.66
GGT U/L	59 [29-112], 690	47.5 [25.8-95], 124	0.05
BDG pg/mL	114 [31-501], 437	78 [40-172.5], 39	0.28
Bacteraemia	204 (28.4%)	22 (16.8%)	**0**.**008**
**CXR**	** n (%)**	**n (%)**	
Multilobar consolidation	244 (39.6%)	50 (43.9%)	0.45
Bilateral GGO	216 (30.1%)	16 (12.2%)	**<0**.**001**
Lobar consolidation	99 (16.1%)	41 (36.0%)	**<0**.**001**
Nodules	42 (6.8%)	5 (4.4%)	0.45
Pleural effusion	16 (2.6%)	2 (1.8%)	0.84

Statistical analysis using Fisher's Exact test and Mann-Whitney U test.

Abbreviations: ALP: alkaline phosphatase; ALT: alanine transaminase; AST: aspartate transaminase; BDG: (1-3)-β-D-glucan; CRP: C-reactive protein; CXR: chest radiograph; GGO: ground glass opacities GGT: gamma-glutamyl transferase; Hb: haemoglobin; Hct: haematocrit; IQR: interquartile range; MAP: mean arterial pressure; n: number; PLWH: People living with HIV; SOI: severity of illness; %: percentage; Bold number indicate significant P values.

The haemoglobin level, white cell and platelet counts, and serum sodium, potassium and albumin, were significantly lower in PLWH, whereas total protein and aspartate transferase (AST) were higher. The predominant radiological finding was multilobar consolidation in both groups, although more HIV-negative patients had lobar consolidation (41 (31.3%) versus 99 (13.8%); P < .001), and a higher proportion of PLWH had bilateral ground glass opacities (216 (30.1%) versus 16 (12.2%); P < .001).

PLWH were significantly less likely to have a microorganism identified but were more likely to have a polymicrobial infection (Table 3). MTB followed by SP were the most common pathogens in both groups, with no significant differences. Among the monomicrobial infections (Table 4), *Staphylococcus aureus* (SA) was more common in the HIV-negative cohort (8 (6.1%) infections versus 16 (2.2%); P = .03), while PJP occurred only in PLWH (35 (4.9%)) (Table 4). Furthermore, more PLWH had bacteraemia (204 (28.4%) versus 22 (16.8%); P = .008) (Table 2). More respiratory viruses were detected in HIV-negative patients; however further statistical analysis was not performed as numbers were small (Table 4).

**Table 3.** Distribution of Monomicrobial and Polymicrobial Infections among HIV-Positive and HIV-Negative Patients with SCAP (849 Patients).

Microbial aetiology	PLWH, n (%)	HIV-ve, n (%)	P value+
No microorganism	303 (42.2%)	68 (51.9%)	**0**.**049**
Monomicrobial infection	329 (79.3%)	58 (92.1%)	**0**.**03**
Polymicrobial infections	86 (20.7%)	5 (7.9%)	

^+^Fisher's Exact test

Abbreviations: HIV: Human Immunodeficiency Virus; PLWH: people living with HIV; n: number; -ve: negative; %: percentage; Bold number indicate significant P values.

**Table 4.** Aetiology of Monomicrobial Infections among HIV-Positive and HIV-Negative Patients with SCAP (42 l Patients).

Microbial aetiology	PLWH, n (%)	HIV-ve, n (%)	P value
***Mycobacterium* spp.**			
MTB,120	106 (32.2%)	16 (27.6%)	0.58
MAC, 1	1 (0.3%)	0 (0.0%)	–
**Gram** **+** **ve pathogens**			
*Streptococcus pneumoniae*, 97	84 (25.5%)	13 (22.4%)	0.73
*Staphylococcus aureus*, 24	16 (2.2%)	8 (6.1%)	**0.03**
*Streptococcus pyogenes,*2	1 (0.3%)	1(1.7%)	–
*Enterococcus faecalis*,1	1 (0.3%)	0 (0.0%)	–
**Gram-ve pathogens**			
*Haemophilus influenzae,* 2	2 (0.6%)	0 (0.0%)	–
Enterobacterales			
* Klebsiella pneumoniae*, 24	18 (5.5%)	6 (10.3%)	0.26
* Escherichia coli*, 9	8 (2.4%)	1 (1.7%)	–
* Enterobacter* spp., 6	5 (1.5%)	1 (1.7%)	–
* Salmonella* spp., 2	2 (0.6%)	0 (0.0%)	–
* Citrobacter freundii.,* 1	1 (0.3%)	0 (0.0%)	–
Other Gram-ve			
*Pseudomonas aeruginosa,* 15	12 (3.7%)	3 (5.2%)	–
*Acinetobacter* spp., 7	6 (1.8%)	1 (1.7%)	–
*Burkholderia cepacia*, 1	1 (0.3%)	0 (0.0%)	–
*Stenotrophomonas maltophilia*, 1	1 (0.3%)	0 (0.0%)	–
**So called “Atypical” pathogens**			
*Chlamydia pneumoniae*,1	0 (0.0%)	1 (1.7%)	–
**Viruses,** 31	24 (7.3%)	7 (12.1%)	0.29
VZV, 15	14 (4.3%)	1 (1.7%)	–
CMV, 1	1 (0.3%)	0 (0.0%)	–
Measles, 4	4 (1.2%)	0 (0.0%)	–
Respiratory viruses, 11	5 (1.5%)	6 (10.3%)	–
**Fungi**			
PJP, 35	35 (10.6%)	0 (0.0%)	**–**
*Candida albicans*, 2	2 (0.6%)	0 (0.0%)	–
*Cryptococcus neoformans*, 1	1 (0.3%)	0 (0.0%)	–
*Saccharomyces cerevisiae*, 1	1 (0.3%)	0 (0.0%)	–
*Stephanoascus ciferii,* 1	1 (0.3%)	0 (0.0%)	–

Statistical analysis using Fisher's Exact test.

Abbreviations: CMV: Cytomegalovirus; HIV: Human Immunodeficiency Virus; KP: *Klebsiella pneumoniae*; MAC: *Mycobacterium avium intracellulare;* MTB: *Mycobacterium tuberculosis*; PJP: *Pneumocystis jirovecii*; PLWH: people living with HIV; n: number; Respiratory viruses: Influenza virus (A, H1N1, B, respiratory virus screen); spp.: species; VZV: Varicella zoster virus; +ve: positive; -ve: negative; %: percent; Bold numbers indicate significant p values.

There were similar proportions of drug-resistant pathogens among PLWH and HIV-negative patients (22 (3.1%) versus 4 (3.1%), respectiv­ely) and although all drug-resistant MTB infections were in PLWH, numbers were too small to obtain statistically meaningful values.

There were no differences in the use of mechanical ventilation (MV) (invasive (IMV) or non-invasive (NIV)), dialysis, inotropic support, and corticosteroids in PLWH compared with the HIV-negative cases.

On univariate analysis (Supplementary Table 1), higher mean arterial pressure, PF ratio, and CRP levels were associated with a lower mortality, as were the presence of infection with *S. pneumoniae*, and lobar consolidation on chest radiograph. Higher Apache II and CURB-65 scores, as well as the presence of GGO on chest radiograph, and the need for ventilation and inotropic support (and trend for need for dialysis (P = .067)) were associated with a higher mortality. On univariate analysis, survival was lower in PLWH (354 (49.3%) compared to 80 (61.1%)) in HIV-negative cases (odds ratio (OR), 1.71; 95% CI, 1.08-2.70; P = .021).

A multivariate model was built as described in the methodology, which included all variables presented in Supplementary Table 2. While some of these variables predicted outcome, others did not, including APACHE II or CURB-65 scores. HIV-positivity per se was not noted to be a predictor of mortality (OR 1.111; 95% CI, .881-1.401; P = .373).

Among the PLWH, there were no differences in age, sex, or smoking habits in those who lived compared with those who died. A total of 225 (31.3%) PLWH were on antiretroviral therapy (ART), of which 111 (15.5%) were initiated prior to hospitalization, and 114 (15.9%) during ICU admission, and the proportion of patients who lived and died were similar regardless of whether they were initiated on ART, prior to or during, their ICU stay. The CD_4_ count was higher among patients on ART who lived, and the median level was 59 [IQR 23.5–122] cells/mm^3^ (P = .075) Cardiovascular disease was more common in patients that lived (47 (13.3%) versus 30 (8.2%); P = .03), while there was no difference in the overall presence of other co-morbidities among those who lived and those who died.

Table 5 documents the clinical presentation, laboratory results and chest radiography in PLWH that lived or died. CURB-65 and Apache II scores were significantly higher in non-survivors, whereas PF ratios were lower. Serum potassium and BDG were both significantly higher in PLWH that died than in survivors. BDG was also significantly higher in PLWH that died without documented fungal infections; however, in patients with PJP infection, BDG levels were similar between survivors and non-survivors. CRP, total serum protein, and albumin levels, as well CD_4_ cell counts in those not on ART were lower in the PLWH who died compared with survivors. There were no differences in HIVVL between the two groups. Bilateral GGO were the predominant finding among the patients who died, however, a higher proportion of survivors had lobar or multilobar consolidation on CXR.

**Table 5.** Clinical Presentation, Laboratory Results and Chest Radiography among HIV-Positive Patients who Lived and Died.

Clinical features, median [IQR], n or n (%)	Total HIV	Lived	Died	P value
Temperature (^o^C)	36.9 [36.2-37.7], 699	37.0 [36.4-37.8], 344	36.8 [36-37.5], 355	**0** **.** **014**
Temperature ≤36 °C and/or ≥ 37 °C	236 (33.8%)	119 (34.6%)	117 (33%)	0.71
Confusion	372 (51.8%)	171 (48.3%)	201 (55.2%)	0.08
MAP mm Hg	79.3 [65.8-91.7], 683	82 [69.9-94.7], 344	77.3 [63.5-88], 339	**<0**.**001**
Bacteraemia	204 (28.4%)	101 (28.5%)	103 (28.3%)	0.99
** SOI scores, median, (IQR), n**				
CURB-65	2 [2-3], 692	2 [1-3], 344	3 [2-3], 348	**0**.**003**
Apache II	18 [14-23], 669	17 [14-22], 335	20 [15-24], 334	**<0**.**001**
Apache II > 25	102 (19.85%)	35 (9.9%)	67 (18.4%)	**0**.**002**
PF ratio	150 [96.7-230.3], 675	175 [118.3-251.8], 335	121.1 [82.9-205.5], 340	**<0**.**001**
PF ratio ≤250	533 (74.2%)	250 (70.6%)	283 (77.7%)	**0**.**04**
**Laboratory, median (IQR), n**				
Hb g/dl	10.2 [8.3-11.8], 713	10.2 [8.5-11.9], 353	10.2 [8.2-11.8], 360	0.46
Hct %	0.3 [0.3-0.4], 713	0.3 [0.3-0.4], 353	0.3 [0.3-0.4], 360	0.76
WCCx10^9^/L	9.1 [5.1-14.0], 712	8.6 [5.3-13.6] 352	9.8 [5.0-14.1], 360	0.31
Platelet count ×10^9^/L	206 [118-302.5], 711	205 [130-291], 351	208.5 [101.8-309.3], 360	0.52
Sodium, mmol/dL	135 [131-140], 714	135 [130-140], 353	136 [131-141], 362	0.19
Potassium, mmol/dL	4.3 [3.8-5.0], 714	4.2 [3.7-4.9], 353	4.4 [3.9-5.0], 361	**0**.**009**
Urea mmol/L	11.9 [6.1-23.5], 714	12.2 [6.3-22.4], 353	11.5 [6.0-25.1], 361	0.97
Creatinine umol/L	124 [71-297], 713	118 [73-288], 353	130 [69.8-303.3], 360	0.81
CRP mg/L	195.5 [116-283], 676	215 [121.3-298.8], 338	186.5 [112.3-267.8], 338	**0**.**005**
T protein g/L	64 [54-75.5], 687	67 [56-77], 341	61 [52-73.8], 346	**0**.**0003**
Albumin g/L	25 [21-29], 686	26 [22-30], 341	24 [20-28], 345	**0**.**001**
T bilirubin umol/L	11 [6-23], 690	11 [6-23], 343	11 [6-23.5], 347	0.57
AST U/L	91 [49-202.5], 690	88 [44-177], 343	95 [54-218], 347	0.07
ALT U/L	48 [25-94.3], 688	47.5 [25-96.5], 340	48.5 [25.8-91.3], 348	0.95
ALP U/L	99 [70-152], 688	97 [65 -151], 341	100 [74.5-152], 347	0.23
GGT U/L	59 [29-112], 690	62 [31-116.3], 342	57.5 [29-112], 348	0.65
BDG pg/mL	114 [31-501], 437	60.5 [31-297], 208	315 [51-501], 229	**<0**.**001**
CD_4_ count cells/mm^3^	42 [14-108], 574	53.5 [19.5-137.5], 314	27.5 [10-81], 260	**<0**.**001**
CD_4_ count cells/mm^3^ ART	48 [16-103], 87	59 [23.5-122], 51	39.5 [12.3-85.8], 36	0.075
CD_4_ count cells/mm^3^ no ART	40 [14-105], 479	53 [19-139], 257	27 [10-78], 222	**<0**.**001**
VL copies/mL	127000 [2675-988939.2], 194	101905 [1400-925757], 109	146000 [9200-1010000], 85	0.44
CXR, n (%)	617 (85.9%)	317 (89.6%)	300 (82.6%)	
ML consolidation	244 (34.0%)	137 (38.7%)	107 (29.4%)	**0**.**01**
Bilateral GGO	216 (30.1%)	82 (23.2%)	134 (36.8%)	**<0**.**001**
Lobar consolidation	99 (13.8%)	64 (18.1%)	35 (9.6%)	**0**.**001**
Nodules	42 (5.8%)	25 (7.1%)	17 (4.7%)	0.23
Pl effusion	16 (2.2%)	9 (2.5%)	7 (1.9%)	0.76

Statistical analysis using Fisher's Exact and Mann-Whitney U test.

Abbreviations: ALP: alkaline phosphatase; ALT: alanine transaminase; AST: aspartate transaminase; BDG: beta-D-glucan; CD_4_: cluster of differentiation 4; CRP: C- reactive protein; CXR: chest radiograph; GGO: ground glass opacities; GGT: gamma-glutamyl transferase; Hct: haematocrit; Hb: haemoglobin; IQR: interquartile range; MAP: mean arterial pressure; ML: multilobar; n: number; PJP: *Pneumocystis jirovecii* pneumonia; pl: pleural; n: number; SOI: severity of illness; temp: temperature; T: total; VL: viral load; WCC: white cell count; %: percent.

**Bold numbers indicate significant p values.**

Supplementary Table 3 shows the comparison of outcomes and mortality among PLWH. Length of stay (LOS) in ICU was similar between PLWH that lived versus those that died. Slightly fewer PLWH survived ICU and survived to hospital discharge.

Supplementary Table 4 shows a comparison of outcomes and mortality comparing PLWH and those that were HIV-negative. There is a statistically significant relationship between HIV status and ICU survival.

Supplementary Table 5 describes the monomicrobial infections in PLWH and their effect on mortality. No difference in survival was noted among PLWH when no identifiable pathogen was documented. SP infection was more common in survivors; however, PJP infections and *Acinetobacter* spp., infections were higher in non-survivors. There were no differences in the frequency of MTB, Gram-negative or viral infections between the two groups.

A higher proportion of PLWH that required MV died; however, more patients who received NIV survived compared to those on IMV. Moreover, there was no difference in survival in patients on NIV compared to no form of MV. Inotrope use was higher in patients that died, but there was no difference in corticosteroid use or dialysis between the two groups (Supplementary Figure 1).

## Discussion

This study described a large cohort of patients with SCAP, of whom a significant proportion were PLWH, in a geographical region with a high HIV burden. The cohort was young, determined mainly by the significantly younger age of PLWH. There were more females among PLWH, with more males in the HIV-negative group. MTB was the most common pathogen in both groups, followed by SP. The mortality of PLWH was higher on univariate analysis (OR 1.71; 95% CI 1.08-2.70; P = .02), but HIV per se did not remain a predictor of mortality when corrected for covariates on multivariable analysis.

The younger age, largely due to PLWH, is similar to the findings of other studies from South Africa,^12,32,43–45^ as well as some international studies with significant numbers of PLWH,^3,35^ but differs from other international studies of SCAP, in which patient age was considerably higher.^46–50^ The younger age of the current cohort is representative of the South African population, which has a median age of 27.6 years (62.2% between 15-60 years), with PLWH comprising 18.7% of those aged 15–49 years.^51^

The frequency of the different comorbidities varies in studies of SCAP, most likely reflecting local disease patterns, the average age of the population, and their habits.^48^ For example, the proportion of PLWH in this cohort was much higher than in other studies.^46^ The reason for the high number reflects the demographics of the South African population, which has the largest number of PLWH in the world, with 13.7% of the general population infected, and up to 20% of those aged between 15 and 49 years.^52^ Only 15.5% of PLWH in this study were using ART prior to admission; unfortunately, this study was not able to determine why this was the case, it is important to recognise that the ART program in South Africa was only initiated in 2004, and initially only among patients with very low CD4 cell counts. Since this study began in early 2007, this may have accounted for some of these findings in the earlier years. A study performed more recently at CMJAH did elucidate additional reasons.^53^

There were an almost equal number of male and female patients in the current study, which differs from several other international SCAP studies which had a higher proportion of males.^7,34,46,47,49,50,54,55^ This was due to PLWH being more commonly female and more females are living with HIV in South Africa,^51^ especially in the 15–24-year-old age group.^52^

The original validation of CURB-65^39^ excluded PLWH, those with suspected pulmonary Tb (PTB), and studied an older population. The median CURB-65 score in the current cohort was higher in patients who died (P = .002). However, PLWH tended to have lower CURB-65 scores compared to those without HIV (P = .06), although all were cases admitted to ICU. This finding is consistent with other CAP data, in which PLWH hospitalised with CAP also had lower CRB-65 (CURB-65 excluding urea) and pneumonia severity index (PSI) scores.^18^ Possible reasons include the younger age of PLWH, and the infrequent finding of confusion and hypotension in patients with PJP.^56^ As such, the CURB-65 score is an imprecise tool for assessing severity in this population.

The median Apache II score was 18 [IQR 14-23] in PLWH, with a predicted mortality of 24% [IQR 15%-40%], lower than the mortality in this study. It has previously been shown that the Apache II score underestimates mortality in patients with SCAP requiring IMV,^57^ and/or in those with a low total lymphocyte count, pneumonia or sepsis.^58^ In this study, however, the Apache II score was higher in patients that died and in PLWH, and it was also an independent predictor of mortality on MVA in the original study,^37^ similar to other studies of SCAP.^47,57^ Furthermore, the PF ratio was shown to be an independent predictor of mortality (both univariate and multivariable analysis) as was noted in the original study.^37^ While those who died had a lower mean arterial pressure (MAP) and more inotrope use, there were no differences in these parameters between PLWH and HIV-negative cases.

Albumin levels were significantly lower in PLWH than HIV-negative patients. This may reflect pneumonia severity,^59^ or the large proportion of PLWH, the majority of whom were not on ART, as albumin levels in PLWH are lower pre-ART and correspond with weight and CD4 cell count.^60^

Similar to other SCAP studies in both PLWH and HIV-negative patients,^3,47,54,55,57,61^ multilobar consolidation was the predominant radiological finding (34.7%) with a similar incidence in both groups. Ground-glass opacities were more common in PLWH and were significantly associated with PJP infection and associated with a higher mortality. Patients with lobar consolidation, more common in HIV-negative patients and in those with SP and MTB infection were, regardless of HIV status, more likely to live, similar to other data from Pune.^34^

An identifiable microbial cause was found in 478 (56.3%) of cases, more commonly in the HIV-negative group. This is lower than other studies,^35,47,62^ probably related to their use of more comprehensive diagnostic techniques.

The current study found that MTB was the most common organism, regardless of HIV status, both among mono- and polymicrobial infections. This reflects the high burden of tuberculosis in the community which is strongly related to HIV infection.^2,63^ MTB had no impact on mortality, even in PLWH, although a high mortality has previously been noted in pneumonia patients admitted to ICU.^64^

SP was the second most common bacterium isolated in both groups. This differs from studies, in which the pneumococcus is the predominant pathogen,^65^ although, most SCAP studies have excluded patients with Tb and/or PLWH.^7,34,43,46,49,50^ On univariable analysis, patients with pneumococcal infection had a lower risk of mortality even in PLWH. SA infections, although well described in PLWH,^1,66^ were more common in the HIV-negative group.

There were a similar number of infections due to Gram-negative enteric bacteria (GNEB) in PLWH compared with the HIV-negative group. However, in another SCAP study, there were more in PLWH relative to the HIV-negative group (20% vs 0%; P < .001),^35^ and in yet another study, a high incidence of *Pseudomonas aeruginosa* (PA) was noted in PLWH with SCAP (17.9%), which was associated with a mortality of 20.6%.^67^

Varicella zoster virus (VZV) infections were the most common viral infection, with most in PLWH, of whom most survived (10 (71.4%) lived and 4 (28.6%) died). Respiratory viruses were more common in HIV-negative patients; however, there were no differences in mortality overall. All PJP infections occurred in PLWH and were responsible for 10.6% of all monomicrobial infections.

A total of 10.5% of patients had polymicrobial infections, the third most common aetiology, occurring more commonly in PLWH. This was similar to a cohort of PLWH with CAP,^3^ in which 11.5% of infections were polymicrobial (after SP and PJP). In the current study, there was no increased mortality among patients with polymicrobial infections.

Although 26.6% of infections in the current study were bacteraemic, more common among PLWH (P = .008), it was not associated with mortality in PLWH. Park *et al*, reported a trend towards more bacteraemia in patients with SCAP compared to those without (14% vs 8%; P = .07).^35^

In the ICU, 726 patients (85.5%) were ventilated, (predominantly IMV), with a similar proportion in PLWH and HIV-negative patients, and this was higher than in other SCAP studies.^46,49,54,57,68^ There was also an increased mortality where IMV was required, which is compatible with another cohort of PLWH with CAP, in which 15.4% required IMV and had an increased 30-day mortality.^3^ Intravenous CS were used in most patients in the current study (650 (76.6%)) of which some may have been for adjunctive therapy of PJP; however, there was no mortality difference noted in those that did or did not receive CS overall. Other studies have noted benefit with the use of CS in SCAP.^26,69–72^

Fifty percent of all patients required inotropic support, associated with increased mortality, as noted in other studies of SCAP.^49,50,73^ There were no differences in the use of IMV or NIV, dialysis, or inotropic support, between PLWH and HIV-negative cases.

The ICU LOS in the current study was increased in patients on IMV and who required renal dialysis and was similar regardless of survival (P = .072). Furthermore, LOS was similar between PLWH and HIV-negative patients. Other studies of PLWH with CAP have noted a difference in hospital LOS depending on the pathogen isolated (10 days with PJP infection vs 7 with bacterial CAP, P = .03).^3^

Overall ICU mortality was 48.9%, similar to SCAP studies from Johannesburg (47.4%)^43^ and in Pretoria, South Africa,^12^ but higher than in other SCAP studies.^34,46,48–50^ Although PLWH had a higher risk of mortality than their HIV-negative counterparts on univariate analysis, with a similar trend on MVA, HIV was not found to be a predictor of mortality when considered in the context of other covariables on multivariable analysis. Cilloniz *et al*, reported a 30-day mortality of 6.6% in PLWH with CAP, which was lower with a bacterial aetiology compared to PJP infection (5.5% vs 14.3%, P = .03), but higher than in HIV-negative patients.^3^ Cordero *et al,* also reported an attributable mortality of 13.1% for PLWH with SCAP, and this was associated with radiological progression, septic shock and CD_4_ count <100 cells/mm^3^.^67^ Within our cohort, the median CD_4_ count was 44 (95% CI, 14-110.5) cells/mm^3^, and the higher mortality noted may reflect the larger proportion of patients with HIV co-infection with MTB, PJP and other opportunistic infections.

This study appears to be the largest study of SCAP in PLWH. Nevertheless, it has potential limitations. First, it was a single-centre study from South Africa, and so the findings may not be generalizable. Second, it only included cases with SCAP admitted to ICU, and so the findings are only pertinent to this category of patients. Third, the study was a record review and since there was limited access to electronic medical records, much of the data had to be retrieved from stored files, some of which were incomplete and, because they did not allow confirmation of the diagnosis of CAP, these cases were excluded, with the consequence that there may have been a significant underestimation of the number of cases of SCAP. Fourthly, due to initial unavailability, and subsequent limited use of the more modern microbial diagnostic techniques because of resource constraints, it is likely that a proportion of pathogens, specifically viruses and atypical microorganisms, may have been undiagnosed, although putative pathogens were documented in 56.3% (n = 478) of cases. Lastly, it was concerning to note in this cohort of patients with SCAP, as mentioned previously, that the frequency of antiretroviral therapy (ART) use was extremely low, with associated low CD4 cell counts and high viral loads in most of the patients, clearly significantly contributing to their poor outcome. This may have been different in the presence of a highly successful ART programme as currently exists in South Africa.^74^ However, the initial approach of the South African Government to HIV in the first decade of its occurrence has previously been described as being denialist, and it was only in the later years, certainly post 2008, that the ART programmes expanded aggressively.^74^ Nevertheless, there still remain a number of individuals who are unaware of their HIV status, and therefore are not on ART, who only become identified once they present with a significant infectious illness, such as SCAP.

## Conclusions

This was a study of 849 patients with SCAP admitted to ICU at a tertiary referral hospital and appears to be the largest ICU cohort of patients with SCAP. The study documented various similarities and differences between the two groups of patients. The ICU mortality of 48.9% was very high and while PLWH appeared to have a higher risk of mortality than their HIV-negative counterparts, HIV per se was not a predictor of mortality on multivariable analysis.

## Supplemental Material

sj-docx-1-jic-10.1177_08850666251359546 - Supplemental material for Severe Community-Acquired Pneumonia: Impact of HIV on Clinical Presentation, Microbiological and Laboratory Findings, and Outcome Supplemental material, sj-docx-1-jic-10.1177_08850666251359546 for Severe Community-Acquired Pneumonia: Impact of HIV on Clinical Presentation, Microbiological and Laboratory Findings, and Outcome by JP Venturas, A Titus, GA Richards and C Feldman in Journal of Intensive Care Medicine

## References

[1] Benito N Moreno A Miro JM Torres A . Pulmonary infections in HIV-infected patients: An update in the 21st century. Eur Respir J. 2012;39(3):730–745. 21885385 10.1183/09031936.00200210

[2] Aston SJ Ho A Jary H , et al. Etiology and risk factors for mortality in an adult community-acquired pneumonia cohort in Malawi. Am J Respir Crit Care Med. 2019;200(3):359–369. 30625278 10.1164/rccm.201807-1333OC PMC6680311

[3] Cilloniz C Torres A Polverino E , et al. Community-acquired lung respiratory infections in HIV-infected patients: Microbial aetiology and outcome. Eur Respir J. 2014;43(6):1698–1708. 24525448 10.1183/09031936.00155813

[4] Brown J Lipman M . Community-Acquired pneumonia in HIV-infected individuals. Curr Infect Dis Rep. 2014;16(3):397. 24562542 10.1007/s11908-014-0397-x PMC7088745

[5] Almeida A Boattini M . Community-Acquired pneumonia in HIV-positive patients: An update on etiologies, epidemiology and management. Curr Infect Dis Rep. 2017;19(1):2. 28160220 10.1007/s11908-017-0559-8

[6] Cillóniz C Torres A Manzardo C , et al. Community-Acquired pneumococcal pneumonia in virologically suppressed HIV-infected adult patients: A matched case-control study. Chest. 2017;152(2):295–303. 28302496 10.1016/j.chest.2017.03.007

[7] Cilloniz C Ferrer M Liapikou A , et al. Acute respiratory distress syndrome in mechanically ventilated patients with community-acquired pneumonia. Eur Respir J. 2018;51(3):1702215. doi:10.1183/13993003.02215-2017 29545274

[8] Urama U Enweani I Oshim I Okeke-Nwolisa B Godsplan U . Microbiological profile of respiratory tract infections among HIV sero-positive subjects attending nnamdi azikiwe university teaching hospital nnewi, Nigeria. Amer J Med and Med Sci. 2018;8(3):37–42.

[9] Cillóniz C Ielpo A Torres A . Treating HIV-positive/non-AIDS patients for community-acquired pneumonia with ART. Curr Infect Dis Rep. 2018;20(11):46. 30203191 10.1007/s11908-018-0652-7

[10] Solomon FB Angore BN Koyra HC Tufa EG Berheto TM Admasu M . Spectrum of opportunistic infections and associated factors among people living with HIV/AIDS in the era of highly active anti-retroviral treatment in dawro zone hospital: A retrospective study. BMC Res Notes. 2018;11(1):604. 30126450 10.1186/s13104-018-3707-9 PMC6102885

[11] Alem K . Prevalence of bacterial pneumonia among HIVSeropositive patients in East Africa: Review. Cogent Medicine. 2021;8(1):2015883.

[12] Ueckermann V van Rensburg L J Pannell N Ehlers M . Characteristics and outcomes of patients admitted to a tertiary academic hospital in Pretoria with HIV and severe pneumonia: A retrospective cohort study. BMC Infect Dis. 2022;22(1):548. 35705920 10.1186/s12879-022-07522-z PMC9202192

[13] Tilahun M Gebretsadik D Seid A , et al. Bacteriology of community-acquired pneumonia, antimicrobial susceptibility pattern and associated risk factors among HIV patients, northeast Ethiopia: Cross-sectional study. SAGE Open Med. 2023;11:20503121221145569. 36632083 10.1177/20503121221145569 PMC9827525

[14] Schleenvoigt BT Ankert J Barten-Neiner G , et al. Pathogen spectrum of community acquired pneumonia in people living with HIV (PLWH) in the German CAPNETZ-cohort. Infection. 2024;52(1):129–137. 37423969 10.1007/s15010-023-02070-3 PMC10811115

[15] Assefa A Woldemariam M Aklilu A , et al. Typical pneumonia among human immunodeficiency virus-infected patients in public hospitals in southern Ethiopia. PLoS One. 2024;19(7):e0307780. 10.1371/journal.pone.0307780 PMC11288459 39078837

[16] Kc R Adhikari S Bastola A , et al. Opportunistic respiratory infections in HIV patients attending sukraraj tropical and infectious diseases hospital in Kathmandu. Nepal. HIV AIDS (Auckl). 2019;11:357–367. 31920403 10.2147/HIV.S229531 PMC6939395

[17] Christensen D Feldman C Rossi P , et al. HIV Infection does not influence clinical outcomes in hospitalized patients with bacterial community-acquired pneumonia: Results from the CAPO international cohort study. Clin Infect Dis. 2005;41(4):554–556. 16028168 10.1086/432063

[18] Malinis M Myers J Bordon J , et al. Clinical outcomes of HIV-infected patients hospitalized with bacterial community-acquired pneumonia. Int J Infect Dis. 2010;14(1):e22–e27. 10.1016/j.ijid.2009.03.001 19586789

[19] Chew KW Yen IH Li JZ Winston LG . Predictors of pneumonia severity in HIV-infected adults admitted to an urban public hospital. AIDS Patient Care STDS. 2011;25(5):273–277. 21488749 10.1089/apc.2010.0365 PMC4056456

[20] Almeida A Almeida AR Castelo Branco S Vesza Z Pereira R . CURB-65 and other markers of illness severity in community-acquired pneumonia among HIV-positive patients. Int J STD AIDS. 2016;27(11):998–1004. 26394997 10.1177/0956462415605232

[21] Madeddu G Laura Fiori M Stella Mura M . Bacterial community-acquired pneumonia in HIV-infected patients. Curr Opin Pulm Med. 2010;16(3):201–207. 20154625 10.1097/MCP.0b013e3283375825

[22] Feldman C Glatthaar M Morar R , et al. Bacteremic pneumococcal pneumonia in HIV-seropositive and HIV-seronegative adults. Chest. 1999;116(1):107–114. 10424512 10.1378/chest.116.1.107

[23] Sadashivaiah J Carr B . Severe Community Acquired Pneumonia. Continuing Education in Anaesthesia. Critical Care and Pain. 2009;9(3):87–91.

[24] Liapikou A Cilloniz C . Severe community-acquired pneumonia: Severity and management. Community Acquired Infection. 2015;2(1):3–7.

[25] Morgan AJ Glossop AJ . Severe community-acquired pneumonia. BJA Educ. 2016;16(5):167–172. 32288942 10.1093/bjaed/mkv052 PMC7104960

[26] Boyles TH Brink A Calligaro GL , et al. South African Guideline for the management of community-acquired pneumonia in adults. J Thorac Dis. 2017;9(6):1469–1502. 28740661 10.21037/jtd.2017.05.31 PMC5506119

[27] Cavallazzi R Ramirez JA . Definition, epidemiology, and pathogenesis of severe community-acquired pneumonia. Semin Respir Crit Care Med. 2024;45(2):143–157. 38330995 10.1055/s-0044-1779016

[28] Cavallazzi R Furmanek S Arnold FW , et al. The burden of community-acquired pneumonia requiring admission to ICU in the United States. Chest. 2020;158(3):1008–1016. 32298730 10.1016/j.chest.2020.03.051 PMC9458541

[29] Martin-Loeches I Torres A Nagavci B , et al. ERS/ESICM/ESCMID/ALAT guidelines for the management of severe community-acquired pneumonia. Eur Respir J. 2023;61(4):615–632. doi:10.1183/13993003.00735-2022 37012080

[30] Ramirez JA Musher DM Evans SE , et al. Treatment of community-acquired pneumonia in immunocompromised adults: A consensus statement regarding initial strategies. Chest. 2020;158(5):1896–1911. 32561442 10.1016/j.chest.2020.05.598 PMC7297164

[31] Chean D Windsor C Lafarge A , et al. Severe community-acquired pneumonia in immunocompromised patients. Semin Respir Crit Care Med. 2024;45(2):255–265. 38266998 10.1055/s-0043-1778137

[32] Nyamande K Lalloo UG John M . TB Presenting as community-acquired pneumonia in a setting of high TB incidence and high HIV prevalence. Int J Tuberc Lung Dis. 2007;11(12):1308–1313. 18034951

[33] Khawaja A Zubairi AB Durrani FK Zafar A . Etiology and outcome of severe community acquired pneumonia in immunocompetent adults. BMC Infect Dis. 2013;13:94. 23425298 10.1186/1471-2334-13-94 PMC3598196

[34] Mane A Gujar P Gaikwad S Bembalkar S Dhamgaye T Risbud A . Aetiological spectrum of severe community-acquired pneumonia in HIV-positive patients from pune, India. Indian J Med Res. 2018;147(2):202–206. 29806610 10.4103/ijmr.IJMR_1590_16 PMC5991133

[35] Park DR Sherbin VL Goodman MS , et al. The etiology of community-acquired pneumonia at an urban public hospital: Influence of human immunodeficiency virus infection and initial severity of illness. J Infect Dis. 2001;184(3):268–277. 11443551 10.1086/322040

[36] Bielosludtseva KO . Features of severe community acquired pneumonia in hiv-infected patients. Medicni Perspektivi. 2014;19(2):53–60.

[37] Venturas JP Richards GA Feldman C . Severe community-acquired pneumonia at a tertiary academic hospital in Johannesburg, South Africa. Respir Med. 2024;234:107823. doi:10.1016/j.rmed.2024.107823 39366492

[38] Mandell LA Wunderink RG Anzueto A , et al. Infectious Diseases Society of America/American thoracic society consensus guidelines on the management of community-acquired pneumonia in adults. Clin Infect Dis. 2007;44(Suppl 2):S27–S72. 10.1086/511159 PMC7107997 17278083

[39] Lim WS van der Eerden MM Laing R , et al. Defining community acquired pneumonia severity on presentation to hospital: An international derivation and validation study. Thorax. 2003;58(5):377–382. 12728155 10.1136/thorax.58.5.377 PMC1746657

[40] Knaus WA . APACHE II score Online [cited 2022 1 April]. Available from: https://www.mdcalc.com/apache-ii-score.

[41] Knaus WA Draper EA Wagner DP Zimmerman JE . APACHE II: A severity of disease classification system. Crit Care Med. 1985;13(10):818–829. 3928249

[42] Department of Health Republic of South Africa. National HIV Testing Services:Policy 2016 [Internet] Prepared by National Department of health. [Cited October 25, 2022] Available from: https://sahivsoc.org/Files/HTS%20Policy%2028%20July%20final%20copy.pdf.

[43] Feldman C Ross S Mahomed AG Omar J Smith C . The aetiology of severe community-acquired pneumonia and its impact on initial, empiric, antimicrobial chemotherapy. Respir Med. 1995;89(3):187–192. 7746911 10.1016/0954-6111(95)90246-5

[44] Feldman C Kallenbach JM Levy H Thorburn JR Hurwitz MD Koornhof HJ . Comparison of bacteraemic community-acquired lobar pneumonia due to Streptococcus pneumoniae and Klebsiella pneumoniae in an intensive care unit. Respiration. 1991;58(5-6):265–270. 1792415 10.1159/000195943

[45] Potgieter PD Hammond JM . The intensive care management, mortality and prognostic indicators in severe community-acquired pneumococcal pneumonia. Intensive Care Med. 1996;22(12):1301–1306. 8986477 10.1007/BF01709542

[46] Vallés J Diaz E Martín-Loeches I , et al. Evolution over a 15-year period of the clinical characteristics and outcomes of critically ill patients with severe community-acquired pneumonia. Med Intensiva. 2016;40(4):238–245. 26391738 10.1016/j.medin.2015.07.005

[47] Quah J Jiang B Tan PC Siau C Tan TY . Impact of microbial aetiology on mortality in severe community-acquired pneumonia. BMC Infect Dis. 2018;18(1):451. 30180811 10.1186/s12879-018-3366-4 PMC6122562

[48] Wongsurakiat P Chitwarakorn N . Severe community-acquired pneumonia in general medical wards: Outcomes and impact of initial antibiotic selection. BMC Pulm Med. 2019;19(1):179. 31619219 10.1186/s12890-019-0944-1 PMC6794881

[49] Paganin F Lilienthal F Bourdin A , et al. Severe community-acquired pneumonia: Assessment of microbial aetiology as mortality factor. Eur Respir J. 2004;24(5):779–785. 15516672 10.1183/09031936.04.00119503

[50] Erdem H Turkan H Cilli A , et al. Mortality indicators in community-acquired pneumonia requiring intensive care in Turkey. Int J Infect Dis. 2013;17(9):e768–e772. 10.1016/j.ijid.2013.03.015 23664334

[51] Statistics South Africa. 2020 Mid-year Population Estimates [Internet] Statistics South Africa [Accessed October 25, 2022] Available from: https://www.statssa.gov.za/?p=13453#:∼:text=South%20Africa's%20mid%20year%20populat ion,released%20by%20Statistics%20South%20Africa.

[52] AVERT. HIV and AIDS in South Africa 2019 [Available from: https://www.avert.org/professionals/hiv-around-world/sub-saharan-africa/south-africa.

[53] Laher AE Richards GA Paruk F Venter WDF . Antiretroviral therapy non-adherence among HIV-positive patients presenting to an emergency department in Johannesburg, South Africa: Associations and reasons. S Afr Med J. 2021;111(8):753–758. 35227356 10.7196/SAMJ.2021.v111i8.15604

[54] Cillóniz C Ewig S Ferrer M , et al. Community-acquired polymicrobial pneumonia in the intensive care unit: Aetiology and prognosis. Crit Care. 2011;15(5):R209. 10.1186/cc10444 PMC3334753 21914220

[55] Li HY Guo Q Song WD , et al. Mortality among severe community-acquired pneumonia patients depends on combinations of 2007 IDSA/ATS minor criteria. Int J Infect Dis. 2015;38:141–145. 26255891 10.1016/j.ijid.2015.07.026

[56] Truong J JV A . Pneumocystis jirovecii Pneumonia. Internet: Treasure Island (FL): StatPearls Publishing; Updated 2023 Jan 21 [Available from: https://www.ncbi.nlm.nih.gov/books/NBK482370/.

[57] Ferrer M Travierso C Cilloniz C , et al. Severe community-acquired pneumonia: Characteristics and prognostic factors in ventilated and non-ventilated patients. PLoS One. 2018;13(1):e0191721. 10.1371/journal.pone.0191721 PMC5784994 29370285

[58] Brown MC Crede WB . Predictive ability of acute physiology and chronic health evaluation II scoring applied to human immunodeficiency virus-positive patients. Crit Care Med. 1995;23(5):848–853. 7736742 10.1097/00003246-199505000-00012

[59] Wiedermann CJ . Hypoalbuminemia as Surrogate and Culprit of Infections. Int J Mol Sci. 2021;22(9):4496. doi:10.3390/ijms22094496 33925831 PMC8123513

[60] Olawumi HO Olatunji PO . The value of serum albumin in pretreatment assessment and monitoring of therapy in HIV/AIDS patients. HIV Med. 2006;7(6):351–355. 16903978 10.1111/j.1468-1293.2006.00391.x

[61] Ewig S Ruiz M Mensa J , et al. Severe community-acquired pneumonia. Assessment of severity criteria. Am J Respir Crit Care Med. 1998;158(4):1102–1108. 9769267 10.1164/ajrccm.158.4.9803114

[62] Sirvent JM de la Torre M C Lorencio C , et al. Predictive factors of mortality in severe community-acquired pneumonia: A model with data on the first 24 h of ICU admission. Med Intensiva. 2013;37(5):308–315. 23669439 10.1016/j.medin.2013.03.003

[63] Bell LCK Noursadeghi M . Pathogenesis of HIV-1 and Mycobacterium tuberculosis co-infection. Nat Rev Microbiol. 2018;16(2):80–90. 29109555 10.1038/nrmicro.2017.128

[64] Levy H Kallenbach JM Feldman C Thorburn JR Abramowitz JA . Acute respiratory failure in active tuberculosis. Crit Care Med. 1987;15(3):221–225. 3469061 10.1097/00003246-198703000-00008

[65] Collaborators GLRI. Estimates of the global, regional, and national morbidity, mortality, and aetiologies of lower respiratory infections in 195 countries, 1990-2016: A systematic analysis for the global burden of disease study 2016. Lancet Infect Dis. 2018;18(11):1191–1210. 30243584 10.1016/S1473-3099(18)30310-4 PMC6202443

[66] Afessa B Green B . Bacterial pneumonia in hospitalized patients with HIV infection: The pulmonary complications, ICU support, and prognostic factors of hospitalized patients with HIV (PIP) study. Chest. 2000;117(4):1017–1022. 10767233 10.1378/chest.117.4.1017

[67] Cordero E Pachón J Rivero A , et al. Community-acquired bacterial pneumonia in human immunodeficiency virus-infected patients: Validation of severity criteria. The grupo andaluz para el estudio de las enfermedades infecciosas. Am J Respir Crit Care Med. 2000;162(6):2063–2068. 11112115 10.1164/ajrccm.162.6.9910104

[68] Ramirez JA Wiemken TL Peyrani P , et al. Adults hospitalized with pneumonia in the United States: Incidence, epidemiology, and mortality. Clin Infect Dis. 2017;65(11):1806–1812. 29020164 10.1093/cid/cix647

[69] Pirracchio R Venkatesh B Legrand M . Low-Dose corticosteroids for critically ill adults with severe pulmonary infections: A review. JAMA. 2024;332(4):318–328. 38865154 10.1001/jama.2024.6096 PMC13475641

[70] Horby P Lim WS Emberson JR , et al. Dexamethasone in hospitalized patients with COVID-19. N Engl J Med. 2021;384(8):693–704. 32678530 10.1056/NEJMoa2021436 PMC7383595

[71] Dequin PF Meziani F Quenot JP , et al. Hydrocortisone in severe community-acquired pneumonia. N Engl J Med. 2023;388(21):1931–1941. 36942789 10.1056/NEJMoa2215145

[72] Sistek CJ Wordell CJ Hauptman SP . Adjuvant corticosteroid therapy for pneumocystis carinii pneumonia in AIDS patients. Ann Pharmacother. 1992;26(9):1127–1133. 1421680 10.1177/106002809202600915

[73] Rello J Rodriguez R Jubert P Alvarez B . Severe community-acquired pneumonia in the elderly: Epidemiology and prognosis. Study group for severe community-acquired pneumonia. Clin Infect Dis. 1996;23(4):723–728. 8909834 10.1093/clinids/23.4.723

[74] Simelela N Venter DWF Pillay Y Barron P . A political and social history of HIV in South Africa. Curr HIV/AIDS Rep. 2015;12(2):256–261. 25929959 10.1007/s11904-015-0259-7

